# Reducing amplification artifacts in high multiplex amplicon sequencing by using molecular barcodes

**DOI:** 10.1186/s12864-015-1806-8

**Published:** 2015-08-07

**Authors:** Quan Peng, Ravi Vijaya Satya, Marcus Lewis, Pranay Randad, Yexun Wang

**Affiliations:** Life Science Research and Foundation, QIAGEN Sciences, Inc., Frederick, Maryland USA

## Abstract

**Background:**

PCR amplicon sequencing has been widely used as a targeted approach for both DNA and RNA sequence analysis. High multiplex PCR has further enabled the enrichment of hundreds of amplicons in one simple reaction. At the same time, the performance of PCR amplicon sequencing can be negatively affected by issues such as high duplicate reads, polymerase artifacts and PCR amplification bias. Recently researchers have made some good progress in addressing these shortcomings by incorporating molecular barcodes into PCR primer design. So far, most work has been demonstrated using one to a few pairs of primers, which limits the size of the region one can analyze.

**Results:**

We developed a simple protocol, which enables the use of molecular barcodes in high multiplex PCR with hundreds of amplicons. Using this protocol and reference materials, we demonstrated the applications in accurate variant calling at very low fraction over a large region and in targeted RNA quantification. We also evaluated the protocol’s utility in profiling FFPE samples.

**Conclusions:**

We demonstrated the successful implementation of molecular barcodes in high multiplex PCR, with multiplex scale many times higher than earlier work. We showed that the new protocol combines the benefits of both high multiplex PCR and molecular barcodes, i.e. the analysis of a very large region, low DNA input requirement, very good reproducibility and the ability to detect as low as 1 % mutations with minimal false positives (FP).

**Electronic supplementary material:**

The online version of this article (doi:10.1186/s12864-015-1806-8) contains supplementary material, which is available to authorized users.

## Background

Over the last few years, next generation sequencing (NGS) has become a widely adopted technology in many aspects of discovery and translational research, because of its ability to acquire sequence information and quantification at the same time [1, 2]. Among many applications using NGS, genomic DNA variant analysis and RNA expression analysis are the most popular ones. The scope of these analyses can be either as wide as the whole genome and transcriptome, or as focused as specific regions and gene panels.

Targeted sequencing is particularly advantageous at achieving very high coverage of the region of interest (ROI) while keeping the cost of sequencing and complexity of data interpretation manageable. Having very high sequencing coverage is especially important for discovering cancer mutations present at low fractions. For example, an average sequencing depth of >1,000 reads is typically required for detecting single nucleotide variants (SNVs) present at 5 % fraction with good confidence [3]. Much higher sequencing depth is needed to detect SNVs at less than 5 % fraction. In RNA analysis, a targeted approach can provide more evidence of low expression transcripts, because in transcriptome sequencing most sequence reads are consumed by mid- and high-abundance transcripts, thus often leaving inadequate coverage of low abundance transcripts [4].

There are multiple ways to enrich a target region before NGS. The most commonly used approaches are 1) hybridization capture from sequencing libraries using target specific probes [5] and 2) PCR amplification directly from sample DNA using target specific primers [6]. Although requiring more effort in up front primer design and chemistry optimization, many people still employ PCR amplicon based enrichment because, in general, the PCR process is easier to handle, requires less overall time, is more specific in terms of target sequence enrichment and can easily accommodate much lower DNA input. With the advent of high multiplex PCR, now hundreds to thousands of amplicons can be simultaneously amplified in one reaction, making the coverage of very large regions convenient [7].

Existing target enrichment, library preparation, and sequencing steps all utilize DNA polymerase and amplification processes, which introduce substantial bias (non-uniform amplification) and artifacts (polymerase errors generating sequence changes not present in the original samples). PCR amplification bias significantly affects quantification accuracy, because final sequence read counts may not accurately represent the relative abundance of original DNA and RNA fragments. Polymerase artifacts generated during the PCR cycles will most likely result in many “false” sequence variants present at low fractions in final sequence reads. These low level “false” variants cause difficulty in identifying real somatic mutations present at very low fraction (e.g. less than 2 %) in the sample. The root cause of these problems is the inability to distinguish the initial sampling of different original molecules from the resampling of the same molecule by primers during the PCR process. Such problems are exacerbated when more PCR cycles are needed to deal with low input DNA or poor quality DNA. PCR amplicon based target enrichment is more prone to these problems than the hybridization capture based enrichment for the following reasons. Random shearing or tagmentation process before hybridization capture creates random and diversified fragment ends, which can be used as a unique identifier for each starting DNA molecule [8]. Such unique identifiers offer a limited ability to keep track of different starting molecules and to remove PCR duplicates and associated amplification artifacts. PCR amplicon based enrichment loses such ability because all starting molecules are enriched with the same sequence ends for a given target specific amplicon.

To mitigate the problems of PCR duplication and biased amplification in NGS analysis, researchers have reported the inclusion of known number of synthetic internal standard molecules to improve the accuracy of NGS quantification [9]. Other approaches involve the use of exogenous molecular barcodes (or molecular tags) [8, 10, 11]. This is not to be confused with sample barcodes commonly used in current NGS workflows. The concept of molecular barcoding is that each original DNA or RNA molecule is attached to a unique sequence barcode. Sequence reads having different barcodes represent different original molecules, while sequence reads having the same barcode are results of PCR duplication from one original molecule. Although molecular barcoding cannot prevent PCR duplication from happening, it provides a nice solution to track duplicates and treat them differently for downstream analysis. By employing molecular barcodes, polymerase artifacts generated during PCR can be distinguished from sequence variants present in original molecules. This barcoding has the potential to increase the detection accuracy for mutations at 1 % fraction or lower by removing low level false positives [8, 12, 13]. The target quantification can also be better achieved by counting the number of unique molecular barcodes in the reads rather than counting the number of total reads, as total read counts are more likely skewed for targets by non-uniform amplification [10, 14, 15].

Several variations of molecular barcodes have been successfully applied in NGS applications. Molecular barcodes have been incorporated into the ligation adapters during the library construction step for genome sequencing [13] and transcriptome sequencing [15]. In another study, barcodes were incorporated into molecular inversion probes for targeted somatic mutation detection [12]. Barcodes can also be incorporated into target specific PCR primers (in the form of a short stretch of random bases) in PCR amplicon sequencing [8, 10], thereby eliminating significant shortcomings in amplicon sequencing as mentioned earlier. In this aspect, so far all reported cases have been related to the amplification of one or a few amplicons by primers containing molecular barcodes, such as the analysis of a viral gene in an HIV resistance study [16], the analysis of 16srRNA gene in a human gut microbiota study [17], and the analysis of IG heavy chain in immune repertoire profiling [18]. As a result, those analyses have all been restricted to only very small regions. Thus, it will be beneficial if molecular barcodes can also be applied in high multiplex PCR amplicon sequencing. In order to accomplish this, some technical hurdles need to be overcome, e.g. how to avoid barcode resampling and how to suppress primer dimers in high multiplex PCR conditions.

We have developed and optimized a high multiplex PCR amplicon sequencing process, which can accommodate hundreds of target specific primers containing molecular barcodes in a single reaction. In addition, the new protocol eliminates the need for ligation-based library construction, by adding sequencing adapters during multiplex PCR amplification. Using this protocol, we have constructed amplicon panels of several sizes to demonstrate: 1) the performance in detecting SNVs at 1 % fraction using admixtures of reference materials from the Coriell Institute 2) the performance in quantifying low abundance RNA transcripts using ERCC spike-in controls; and 3) the ability to enrich large regions and detect unknown somatic mutations in FFPE samples. Our data confirmed the superior performance of counting molecular barcodes over counting sequence reads in high multiplex amplicon sequencing. We show that the new protocol combines the simplicity of PCR amplicon sequencing with the accuracy of molecular barcodes, can provide deep coverage for a very large region, and will be a useful addition to existing target enrichment solutions.

## Results

### Overview of the high multiplex amplicon barcoding protocol and assay design

To design primers for our high multiplex amplicon barcoding protocol, we adopted the “Primer ID” design strategy [16] by inserting a molecular barcode region (random 6 to 12mer) between the 5′ universal sequence and 3′ target specific sequence in one of the two primers for each amplicon. All primers containing the molecular barcode for different amplicons are pooled together (“BC primers”) and all other non-barcoded primers are mixed in a different pool (“non-BC primers”). Because of our goal in high multiplex PCR, each target specific primer sequence is selected to minimize potential cross hybridization with other primers. Specifically, a target primer sequence will be rejected when more than ten bases at its 3′ end will form perfect complementary match with another target primer.

**Fig. 1:**
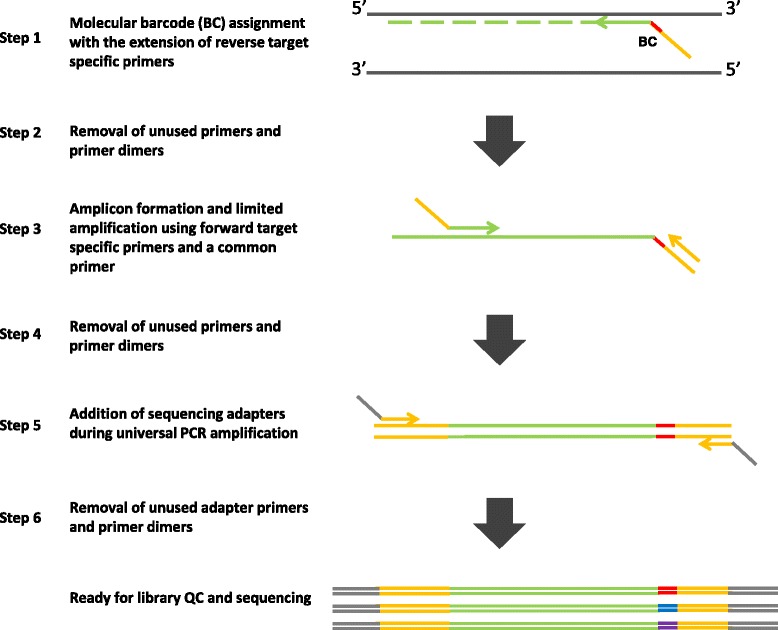
Overview of the high multiplex amplicon barcoding PCR

The workflow is as the following (Fig. 1). 1) The BC primers are annealed to and extended on target DNA. At this step, each DNA molecule containing our target locus will be copied and the resulting copy will have a unique molecular barcode. 2) The unused BC primers are removed through size selection purification. 3) A limited PCR amplification is conducted using the non-BC primers and a universal primer corresponding to the universal sequence in the BC primer. 4) The unused primers are removed from the amplicons. 5) A universal PCR is used to further amplify the material to desired quantity for amplicon sequencing. At this step, platform specific adapter sequences are also introduced to form complete sequencing libraries.

The keys to success in high multiplex amplicon barcoding PCR are minimizing primer dimer formation and controlling resource competition from amplicons of different amplification efficiencies. In general, long primers with universal sequences are more prone to primer dimer amplification in universal PCR. Many different primer dimers may form during the preparation of many barcoded amplicons. Although each dimer may be generated at a low level, they can be amplified together during the subsequent universal amplification to a level that severely hinders the amplification of target amplicons. To avoid this, we physically separated primers with different universal sequences into two pools, to reduce the likelihood of forming primer dimers containing both universal sequences, which would otherwise be amplified during universal PCR. Furthermore, we removed unused BC primers before non-BC primers are added. In our experience, even a minute amount of leftover BC primers can risk dimer formation with non-BC primers, as well as causing a “barcode resampling” problem, i.e. the same DNA input template being associated with multiple molecular barcodes, which defeats the benefits of molecular barcoding. After evaluating several approaches, we found that two-round size selection purification is the most efficient way to remove primer dimer background (Additional file 1: Figure S1). Secondly, because target specific primer extensions were used in limited cycles and our amplification was mostly driven by a pair of universal primers, we minimized the difference in amplification efficiency and competition among many different amplicons.

### Detecting SNVs at very low allelic frequencies

Distinguishing true SNVs in the sample from sequencing or PCR artifacts is usually very challenging because both are often present at very low fractions in the reads. To demonstrate the benefit of molecular barcodes in supressing sequencing artifacts, we first applied our method to detecting SNVs at very low fractions. Following an earlier approach [19] we created a sample containing a set of “known” SNVs at 1–2 % fractions, by mixing DNAs of two well-characterized individuals (NA12878 and NA19129) from the 1,000 Genomes Project. A high-confidence variant set has been developed for NA12878 by the NIST-led “Genome in a Bottle” Consortium [20]. Variant data are also available for NA19129 from the 1,000 Genomes Project.

A total of 741 primers were designed according to our primer design algorithm as described in the Methods section. This DNA Amplicon Panel I covered a 39,231 bp region in the human genome, including 134 high confidence SNVs that were not homozygous reference in NA12878 and were homozygous reference in NA19129. Out of these 134, 118 were heterozygous in NA12878 and 16 were homozygous non-reference in NA12878. With this amplicon panel, we performed target enrichment using 10–80 ng genomic DNA mixtures, following our high multiplex amplicon barcoding protocol. After Illumina MiSeq pair-end sequencing, 4.1 to 5.2 million reads were generated from each sample with a mean coverage depth of at least 8,300x (Table 1).

**Table 1:** Summary of the sequencing runs for in vitro DNA mixtures

Input amount	10 ng	20 ng	40 ng	80 ng	10 ng	80 ng
LA cycles	1	1	1	1	3	3
Total reads	5,161,694	5,029,394	4,181,410	4,568,978	4,612,940	8,718,690
On-target reads	4,449,285	4,226,778	3,528,081	4,051,939	3,591,578	7,704,936
On-target read pairs	2,152,647	2,066,226	1,707,379	1,972,168	1,715,098	3,659,067
Median raw read depth	9,263	8,558	6,454	6,915	7,701	16,275
Mean raw read depth	10,514	10,096	8,332	9,628	8,271	17,635
% Bases >0.2x mean depth	95	94	92	90	95	96
Median consensus read depth	98	195	346	544	209	889
Mean consensus read depth	98	187	336	530	208	839
Mean raw read/consensus read	53	28	13	11	22	16
Median raw read/consensus read	53	26	11	8	20	10
Bases in target region	39,231	39,231	39,231	39,231	39,231	39,231
GIAB high confident region for NA12878	29,343	29,343	29,343	29,343	29,343	29,343
NA12878 unique SNVs	134	134	134	134	134	134
Detected true positives	17	40	76	93	39	114
Detected false positives	0	2	3	5	4	3

**Fig. 2:**
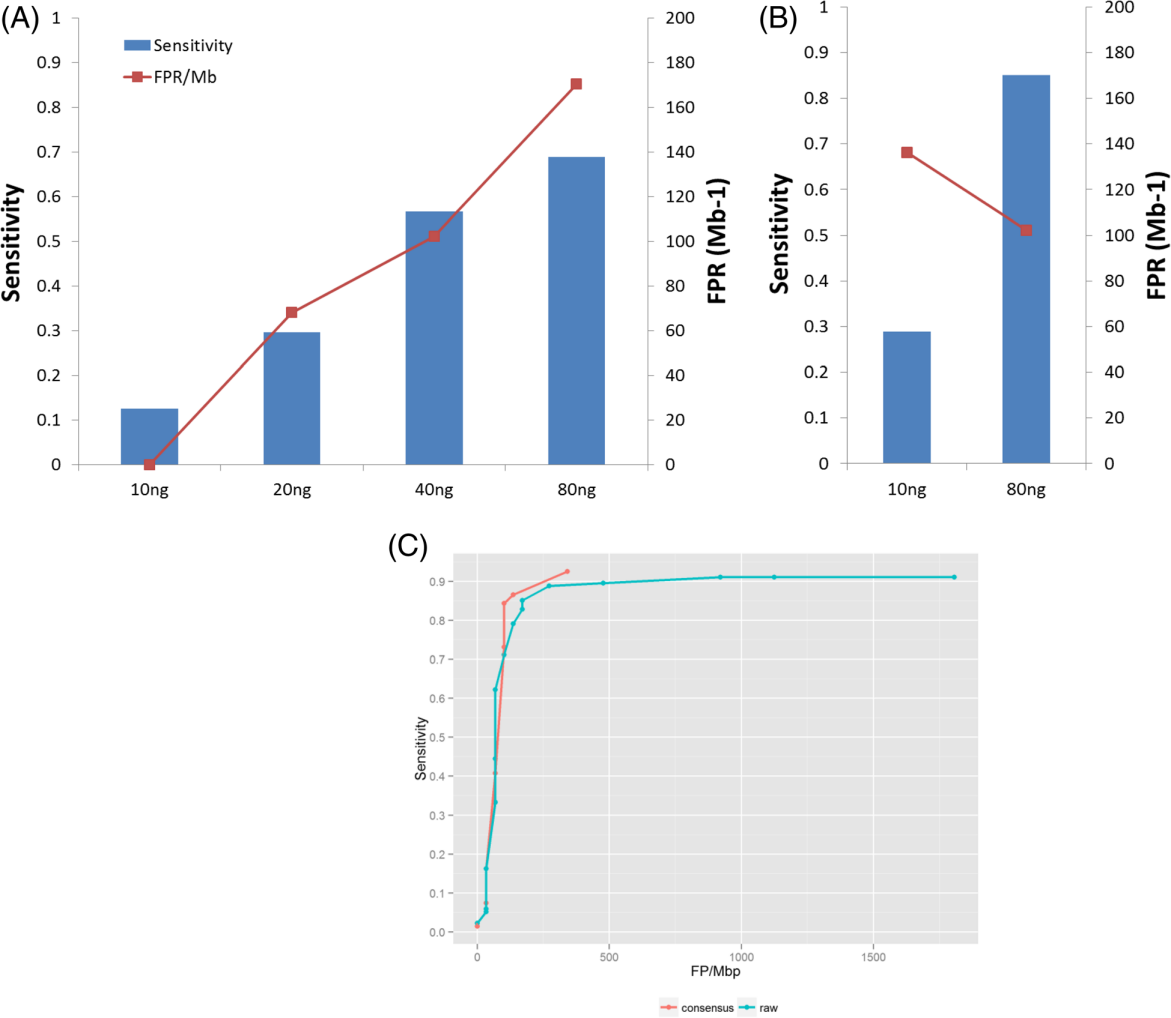
Comparison of sensitivity and false-positive rates for different input DNA amounts. (**a** and **b**) The x-axis represents different input quantity of the DNA admixture. The left y-axis represents detection sensitivity for SNVs at 1–2 % fraction. The right y-axis represents false positive rates (**a**) Performance using the original protocol. (**b**) The sensitivity of SNV detection was significantly higher after adding 3 cycles of limited amplification. (**c**) The ROC curve from 80 ng 3-cycle data with or without using the information of molecular barcodes

Reads from the same amplicon with the same molecular barcode were processed into one consensus read. All consensus reads were aligned to the reference genome and SNVs were identified. For 10, 20, 40 and 80 ng genomic DNA inputs, the mean coverage depths calculated using consensus reads were 98x, 187x, 336x and 530x respectively (Table 1). The number of consensus reads for a chromosomal locus is a reflection of the number of original DNA molecules being enriched for that locus. The higher number of coverage depth based on consensus reads reflected the more genomic DNA copies in the input samples. For SNV detection, 17 out of 134 (expected allelic frequency of 1–2 %) high confidence SNVs were detected (12.7 % sensitivity) in the10ng sample, with no false positives. The sensitivity increased as sample input increased, and reached 68.9 % with 5 false positives when we used 80 ng genomic DNA (Fig. 2a).

These initial results suggested that the greater the fraction of initial DNA molecules being converted to full amplicons by primer pairs, the greater the detection sensitivity that could be achieved. To improve sensitivity, we sought to improve the efficiency in forming full amplicons. One simple solution was to run multiple cycles of non-BC primer annealing/extension, trying to convert as many barcoded DNA fragments as possible into full amplicons. After we changed Step 3 in the protocol from 1 cycle to 3 cycles for 10 ng and 80 ng DNA inputs, our mean coverage depths for consensus reads increased from 98x to 208x and from 530x to 839x respectively (Table 1). As we expected, the sensitivity increased to 29.1 % with four false positives, and to 85.1 % with three false positives, respectively (Fig. 2b).

To compare the performance to that without using molecular barcodes, the raw reads and consensus reads from 80 ng 3-cycle sample were further analysed. Variants were identified using different *tlod* settings in MuTect (Fig. 2c). ROC curves demonstrated that with low or medium sensitivity settings (<70 %), raw reads and consensus reads had similar performance in terms of false positives. With high sensitivity settings (>80 %), using molecular barcodes significantly reduced false positives. These data showed that polymerase and sequencing errors could be major contributor to false positives in variant calling, and using molecular barcodes could efficiently remove those errors and improve data quality. In addition, we believe our current consensus read model and variant calling were not fully optimized and the FPR using molecular barcodes can be further reduced by incorporating more sophisticated statistical methods.

### Measuring low abundance RNA transcripts

Next we evaluated the use of high multiplex amplicon barcoding in targeted quantification of RNA transcripts. To set up this experiment, we used ERCC RNA spike-in control mix as our sample, because each mix contains a defined number of copies for each RNA transcript [21]. The concentrations of 92 polyadenylated transcripts in the mix span 10^6^ fold concentration range. Knowing the sequencing capacity of MiSeq, we excluded 25 transcripts with the highest concentrations from our analysis, and designed 96 amplicons for the remaining 67 transcripts (Additional file 2). For some of the longer transcripts, two amplicons were designed, one close to 5′end and the other close to 3′end. Following the high multiplex amplicon barcoding PCR and MiSeq sequencing, we estimated the abundance of RNA transcripts represented by each amplicon by sequence reads and by counting unique molecular barcodes. We then compared these estimates to the expected amounts in the ERCC RNA mix. We also examined the variability in the first barcode assignment step and in the universal PCR amplification step.

**Fig. 3:**
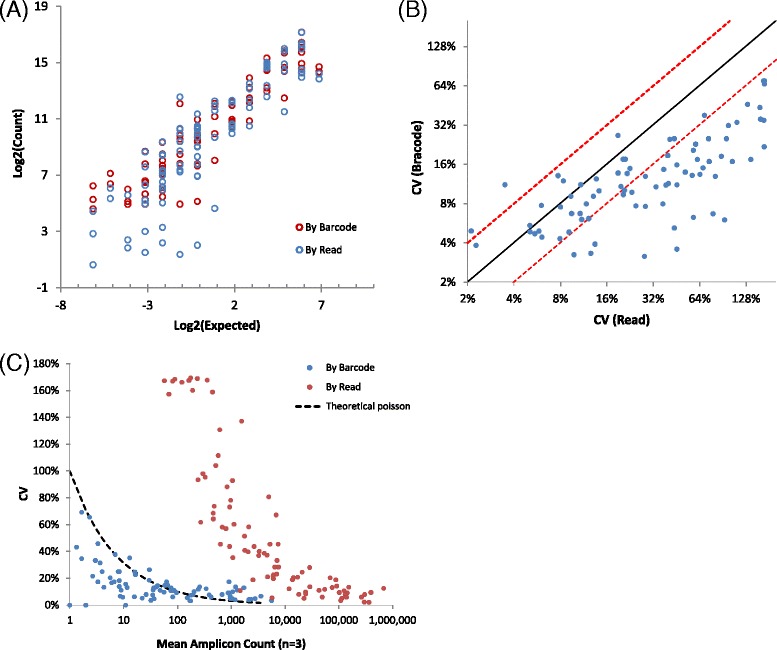
ERCC RNA quantification using amplicon barcoding. (**a**) Correlation between “measured” vs. “expected” numbers for each ERCC RNA transcripts represented by each amplicon. The x-axis represents log2 values of known copies in the ERCC RNA spike-in mix. The y-axis represents log2 values of average barcode or read counts for each amplicon (*n* = 3). Both barcode count and read count from different sequencing runs were first normalized to a mean value of 10,000 for each run before being averaged. (**b**) CV computed on the basis of barcode counts vs. raw read counts. Three independent target enrichment experiments were performed. Solid black line represents diagonal and two red dash lines represent 2-fold intervals. (**c**) CV vs mean plot for both barcode counts and read counts. X-axis represents the mean value for each amplicon on the basis of either barcodes or reads. Corresponding CV is plotted on y-axis. The theoretical Poisson CV is plotted as the black dash line

The measured transcript abundance by each amplicon correlated well with the expected levels (Fig. 3a) overall. Two things are noteworthy. First, the correlations of the “measured” vs. the “expected”, calculated by reads and barcodes, were largely similar for higher abundant transcripts. However, for lower abundant transcripts, the correlation for measurements by barcodes was much better than those by reads, as evidenced by more scattering of read data in the lower left corner. This suggests that the value of using molecular barcodes is more evident for quantifying targets of low abundance. Secondly, the overall correlation using barcodes was still not perfect for our set of amplicons. We postulate that these biases are likely introduced during reverse transcription and initial barcode assignment steps. It is known that reverse transcription efficiency along a RNA transcript can be affected by RNA secondary structures and RNA integrity. Since barcode assignment is accomplished by target specific primers, different primers will also possess different annealing efficiencies. Because these biases are dependent on sequence context, fold change analysis between two samples for the same target may be less affected. In addition, using multiple amplicons sparsely tiling each transcript and using their average value will likely reduce these biases greatly.

In addition, we observed that most measurements using barcodes have much smaller technical noise, assessed by the coefficient of variation (CV), than those using raw sequence reads (Fig. 3b). The technical noise was reduced by about 2.6-fold on average, and for some amplicons, by as high as 10-fold. Most of the technical noise we observed using raw reads were likely the result of universal PCR amplification (Additional file 1: Figure S2). For low abundance transcripts, sampling error could significantly contribute to observed variation. To confirm this, theoretical Poisson distribution CV-vs-mean was plotted (Fig. 3c). CVs for barcodes were very close to the Poisson CV, suggesting that using molecular barcodes enabled us to lower the counting variation close to the theoretical limits set by Poisson sampling error. On the other hand, raw read counts were also influenced by technical noise during multiple PCR cycles and often overestimated the number of original molecules being sequenced, so variations higher than sampling error were observed. Overall our data showed that PCR amplification can be highly stochastic and non-uniform, and counting molecular barcodes instead of reads can efficiently remove PCR amplification variation.

### Application of high multiplex amplicon barcoding protocol to FFPE samples

To demonstrate the scalability of our high multiplex amplicon barcoding protocol and its application in real biological samples, we designed an additional 1,108 primers and combined them with those from DNA Amplicon Panel I to form a larger panel. This DNA Amplicon Panel II was designed to cover all the coding regions of 15 important cancer genes, such as TP53, ATM, EGFR, APC, BRAF, etc. We performed target enrichment experiments using this panel on DNA extracted from commercial FFPE samples. The FFPE samples we used were of vastly different qualities, as measured by GeneRead DNA QuantiMIZE QC assays (Additional file 1: Table S1). Based on our estimates of the PCR amplifiable fractions in the FFPE DNA, we have to adjust the number of universal PCR cycles used in our protocol for poor quality DNAs in order to yield enough material for MiSeq sequencing. The sequencing results showed overall very high percentage of reads on target (>96 %) and very good uniformity (>90 % above 0.2x mean) for all FFPE samples for which we were able to generate enough libraries (Table 2). The presence of molecular barcodes for each amplicon allowed us to look at the actual number of original DNA molecules represented in the raw reads. As expected, the number of original molecules enriched directly correlated with the quality of the FFPE DNA. For example, the total reads for sample T5 were derived from only 33 copies of original DNA on average. This number suggests that it would be very difficult to detect most mutations present below 5–10 % fraction in this FFPE sample under the conditions we used. Such information on the limit of detection per sequencing run would not be available without the use of molecular barcodes. Our data also suggested that deeper sequencing would not help improving the sensitivity in this case. In the end, about 70 to 140 SNVs in our target region were identified from each FFPE sample by using our variant calling pipeline on consensus reads. Particularly, from the two match-paired lung samples we tested, four SNVs, at fractions ranging from 0.7 to 6.0 %, were uniquely identified in the primary tumor. However, we have not yet confirmed the validity of those variants by alternative methods.

**Table 2:** Summary of the sequencing runs for FFPE samples

Sample ID	T5	LN2	LT2	T2
Total reads	1,053,646	11,352,414	13,518,788	9,911,538
On-target reads	1,015,755	11,027,934	13,034,688	9,642,483
On-target read pairs	501,517	5,417,637	6,346,625	4,745,809
Median read depth	820	9,653	11,460	8,420
Mean read depth	1,079	11,534	13,526	10,120
% Bases >0.2x mean depth	90	93	94	93
Median consensus read depth	30	390	908	151
Mean consensus read depth	33	376	891	146
Mean raw read/consensus read	15	14	8	33
Median raw read/consensus read	15	13	7	32
Bases in target region	86,544	86,544	86,544	86,544
Called SNVs	77	129	134	141

## Discussion

High multiplex amplicon PCR is a simple approach to enrich a target region of interest for NGS. It is highly specific and works well for DNA from FFPE sections. However, the multiplex PCR approach has the major drawbacks of no ability to de-duplicate sequence reads and competition among primers with different efficiencies. By incorporating molecular barcodes into the multiplex PCR process and relying on universal amplification, we have avoided these problems and improved the overall performance.

The sensitivity of our method has more room for improvement. The current sensitivity for detecting low fraction variants is limited by low sample input. As the DNA titration experiments suggested, the variant calling sensitivity is positively correlated with the amount of the input DNA. There are approximately 3,300 copies of haploid genomes in 10 ng human genomic DNA. The improved 3-cycle method only captured 208 copies on average, which is about 6 % of the input. For variants at 1 % fraction, on average two copies of the variants were present in the final sequencing data. This may explain why the sensitivity was very low (29 %) for 10 ng genomic DNA. Increasing the efficiency to form full amplicons during initial steps is essential for detecting DNA variants at low fractions when the input is limited. For RNA quantification, this is also important for detecting low abundant transcripts.

There are many steps in the workflow that could result in sample loss and thus offer room for further improvement. Those steps include BC primer extension, BC primer removal, and non-BC primer amplification. According to our estimates, the current conditions for BC primer assignment only captured on average 40 % of input DNA. This step is limited to just one cycle, as “barcode resampling” must be strictly avoided. Using higher concentrations of BC primers could be a way to improve capture efficiency; however it is not always possible, especially with hundreds or thousands of different primers in the reaction. We also know that significant sample loss can happen during the BC primer removal step. The ideal method should be highly efficient for removing unused BC primers and dimers, yet be able to recover as many elongated products as possible to minimize sample loss. Initially we tried to use an enzymatic approach such as Exonuclease I digestion to degrade leftover BC primers. Our study showed that Exo I digestion was not efficient enough, leading to significant amount of primer dimers in the final product (data not shown). Size selection purification in general is more efficient in removing primers but with higher sample loss. By our estimates, probably 50–80 % target DNA was lost during the size selection protocol we used. Improving the size selection process (e.g. using a bead based system) should improve the overall target enrichment efficiency.

The sequence of the molecular barcodes can also affect variant calling performance. We were using random 10mer barcodes in the DNA study. The benefit of completely random barcodes is that they are economical to synthesize. However, since they are completely random, we have only limited ability to distinguish an original barcode from a “mutant” barcode due to PCR or sequencing errors. Those “mutant” barcodes will decrease our ability to remove amplification artifacts in the reads. One way to mitigate this is through barcode clustering, based on the assumption that any “mutant” barcode should come from an ancestor barcode with significantly higher number of reads. The possible number of different barcodes used in our current system is orders of magnitude higher than the number of DNA molecules. In this case, the probability of barcode collision (where two different DNA molecules are tagged with the same barcode) is extremely low. If the edit distance between two observed barcodes is below a certain threshold, it is possible to assume that one of them is a mutated version of the other, and the two barcodes can be merged into a single barcode cluster. Then the barcode cluster is used for building consensus reads and counting molecules. In practice, depending on the application, we can apply different strategies and thresholds for clustering the random barcodes. Overly aggressive clustering can minimize the false-positives, but may also lead to underestimation of the DNA copies and lower sensitivity. On the other hand, if the clustering is not aggressive enough, it can lead to too many false-positive variant calls. Balanced clustering for random molecular barcodes deserves further optimization depending on the application. An alternative way to mitigate barcode errors is to use a mixture of error-correcting barcodes [15]. However, it is practically cost prohibitive to do so for many different primers in high multiplex amplicon PCR.

It is worth noting that in our variant calling example, the false positive rate increased when consensus read depth increased from 10 to 80 ng. We believe this was caused by both higher consensus read depth (mean from 98 to 530) and reduced read coverage of each consensus read (mean from 53 to 11). With higher consensus read depth, more loci gained higher coverage and became callable for MuTect, so both TP and FP increases from 10 to 80 ng. When the read coverage of each consensus read decreases, it’s also possible to get more false positives due to lower quality of consensus reads. When we down sampled the reads in 10 ng (1 cycle) data to 25 % while keeping the consensus read depth about the same, we found that false positives increased from 0 to 2. It is possible that our consensus read modeling can be further optimized so that the lower read coverage of each consensus read has lower impact on false variant calling.

When dealing with low copy number events, i.e. detecting low fraction variants or low abundant RNA transcripts, sampling variation in multiple processes could become a major source of errors affecting data quality. This has been observed and discussed previously in various types of data (PCR, microarray, and sequencing) [9, 22, 23]. In our targeted sequencing application, the use of molecular barcodes (through barcode primers) enables researchers to identify and count only original molecules rather than assuming that each individual sequence read represents a separate original molecule. This practice makes it possible to observe and calculate sampling statistics. Figure 3c, for example, suggests that consensus read counts followed theoretical Poisson distribution while raw read counts were affected by other factors such as PCR amplification bias. This underlines the fact that sampling statistics can be used to greatly improve confidence in both variant calling and RNA transcript counting applications. Taking variant calling as an example and assuming that variant caller needs to see at least 2 variant molecules to make a call, based on negative binomial distribution, around 400 molecules need to be sampled in order to achieve 90 % probability to call variants at 1 % allele fraction. Such sampling statistics can be used to determine the theoretical limit of sensitivity at a given allele fraction and to be able to rule out the existence of an alternative allele with some specified level of confidence. If molecule sampling efficiency is also known, one can calculate the DNA input requirement in order to achieve a given sensitivity at certain allele fractions.

Our protocol is easily scalable to thousands of primers in a single tube and worked well for DNA from FFPE samples. The ability to detect mutations at low fractions is largely affected by the quality of FFPE samples. With the presence of molecular barcode in each read, we now have the ability to estimate the lower limit of variant fractions which can be detected in each FFPE sample. This information could be quite useful in interpreting the significance of negative findings in FFPE samples, as discussed above.

## Conclusions

In summary, we have developed an NGS target enrichment process that integrates molecular barcodes into high multiplex PCR amplicon sequencing. We demonstrated the benefits of molecular barcoding in reducing low level sequencing artifacts, which would otherwise plague the detection of SNVs at very low fractions. Our process was highly reproducible and scalable, and was successfully applied to analyzing a large region of DNA from FFPE sections.

## Methods

### Preparation of in vitro sample mixtures

Human genomic DNA samples of NA12878 and NA19129 were purchased from Coriell Institute. Sample mixtures were created based on the actual amplifiable DNA in each sample, resulting in 2 % of NA12878 DNA mixed in the NA19129 DNA. The resulting DNA mixture contains NA12878 variants present at 1–2 % fraction. Homozygous SNVs unique to NA12878 are at 2 % in the mixture, while heterozygous SNVs are at 1 %. Most of the 134 variants from NA12878 are heterozygous SNVs.

### DNA Amplicon Panel I description

Primers were generated using QIAGEN’s internal primer design algorithm to target an approximately 39 kb region in the human genome. Half of the primers were designed to cover 134 high-confidence SNVs from NA12878. The other half were designed to cover the protein coding regions of three genes: APC, SMAD4 and CTNNB1. To minimize primer dimer in high multiplex PCR, each 3′ target specific sequence was selected to minimize potential cross hybridization with other primers. Specifically, a target sequence would not be selected if more than ten bases at its 3’ end form perfect complementary match with another primer. Primer sequences are provided in Additional file 2. All primers were synthesized by IDT (Coralville, IA).

### DNA Amplicon Panel I enrichment protocol

DNA library was prepared according to the workflow described in the Results section. Briefly, 10 to 80 ng DNA was used in each 10ul reaction, together with 20nM each of BC primer, KOD DNA polymerase and reaction buffer (Toyobo, Japan). The following barcode assignment condition was used: 98 °C for 2 min, 55 °C for 15 min, 65 °C for 15 min, and 72 °C for 7 min. To ensure complete removal of excess BC primers, each sample was purified for two rounds using GeneRead Size Selection Kit (QIAGEN, Germany). The purified DNA was then mixed in 25ul with 20nM each non-BC primer, 4 mM Mg^2+^, 0.45 mM dNTP, 6U HotStarTaq and 1X miScript preamp buffer (QIAGEN, Germany). The reaction was done at following conditions: 95 °C for 15 min; one or three cycles of 95 °C for 15 s, 55 °C for 15 min and 65 °C for 15 min; 98 °C for 15 min. After that, universal adapter primers, new HotStarTaq and buffers were added in proportion to bring the reaction volume to 50ul. The reaction was further incubated at the following conditions: 95 °C for 15 min; 23 (80 ng input) or 26 (other inputs) cycles of 95 °C for 15 s and 60 °C for 2 min. Resulting DNA libraries were purified using GeneRead Size Selection Kit and quantified using GeneRead DNAseq Quantification Kit (QIAGEN, Germany). MiSeq sequencing (pair-end, 2x150bp) was done following manufacturer’s user manual (Illumina, CA). The sequencing reads were processed using QIAGEN’s internal pipeline as described in data analysis section.

### ERCC RNA amplicon enrichment protocol

ERCC RNA Spike-in Control Mix 1 was purchased from Life Technologies (Carlsbad, CA). It was further diluted 1:100 in the background of human normal universal RNA (BioChain, CA). 10 ng total RNA containing the ERCC RNA were reverse transcribed into cDNA using QuantiTect Reverse Transcription kit (QIAGEN, Germany). One fifth of the cDNA was used in the barcode assignment step together with 2nM each BC primer, 16 mM Mg^2+^, 6U HotStarTaq and 1X miScript preamp buffer. The following barcode assignment conditions were used: 95 °C for 15 min, 55 °C for 15 min, 65 °C for 15 min, and 72 °C for 7 min. To ensure complete removal of excess BC primers, reaction was purified in two rounds using GeneRead Size Selection Kit. The purified DNA was then mixed in 25ul with 2nM each non-BC primer, 4 mM Mg^2+^, 0.45 mM dNTP, 6U HotStarTaq and 1X miScript preamp buffer. The reaction was continued at following conditions: 95 °C for 15 min; 20 cycles of 95 °C for 15 s and 55 °C for 5 min; 98 °C for 15 min. After that, universal adapter primers, new HotStarTaq and buffers were added in proportion to bring the reaction volume to 50ul. The reaction was further incubated at the following conditions: 95 °C for 15 min; 26 cycles of 95 °C for 15 seconds and 60C for 2 min. Resulting DNA libraries were purified using GeneRead Size Selection Kit, and quantified using GeneRead DNAseq Quantification Kit.

### FFPE sample preparation

FFPE tissue sections were purchased from BioChain Institute Inc. Samples collected by BioChain were ethically approved by an Institutional Review Board established at BioChain (registered with the Office for Human Research Protections with the registration number of IRB00008283). So samples may be purchased for this study without the requirement for project-specific ethical approval. Each 10um section was used to extract genomic DNA using GeneRead DNA FFPE Kit (QIAGEN, Germany), which contains an enzymatic step to remove cytosine deamination artifacts generated during the formalin fixation process. The quality and amplifiable portion of the extracted DNA were assessed by the GeneRead DNA QuantiMIZE Kit (QIAGEN, Germany). The detailed sample information is provided in Additional file 1: Table S1.

### DNA Amplicon Panel II description

Additional amplicons were designed using the same primer design algorithm used for the DNA Amplicon Panel I, to cover all protein coding regions of another 12 genes: KRAS, TP53, AKT1, ATM, BRAF, FBXW7, PIK3CA, EGFR, ALK, NRAS, BAX and TGFBR2. Those primers were combined with the primers from DNA Amplicon Panel I, resulting in the DNA Amplicon Panel II. The combined panel covers a target region of approximately 87 kb. Primer sequences are provided in Additional file 2.

### DNA Amplicon Panel II enrichment protocol

The amount of FFPE DNA sample used in each reaction was calculated based on the actual amplifiable DNA fragments in each sample, as reported by the GeneRead DNA QuantiMIZE Kit (Additional file 1: Table S1). Barcode assignment and BC primer removal conditions were the same as used for DNA Amplicon Panel I. The purified DNA was then mixed in 25ul with 20nM each non-BC primer, 600nM RS2 primer, 4 mM Mg^2+^, 0.45 mM dNTP, 6U HotStarTaq and 1X miScript buffer. The reaction was continued according to the following conditions: 95 °C for 15 min; two cycles of 95 °C for 15 s and 60 °C for 15 min; eight cycles of 95 °C for 15 s and 60 °C 5 min. The PCR products were purified two round using the GeneRead Size Selection Kit. The purified DNA were further amplified in 25ul using 200nM universal adapter primers, 4 mM Mg^2+^, 0.45 mM dNTP, 6U HotStarTaq and 1X miScript buffer, according to the following conditions: 95 °C for 15 min; 25 to 29 cycles of 95 °C for 15 s and 60 °C for 2 min. The resulting DNA libraries were purified, QC’ed and sequenced as described earlier.

### Barcode extraction

The raw reads were first processed using cutadapt [24]. The universal sequences at the 5′ end of the reads and the possible reverse complements of these sequences at the 3′ ends of the reads were removed using two separate runs of cutadapt. The trimmed reads were then mapped to the genome using BWA [25]. The molecular barcodes were extracted from trimmed reads by using the intended primer locations as reference points and extracting the bases between the 5′ end of the trimmed read and the primer start position in the aligned read. Off-target reads were ignored.

### Barcode clustering

To allow for the possibility of PCR or sequencing error within the barcode regions, we implemented a custom barcode clustering procedure to identify all barcodes that putatively originated from the same initial molecular tag. First, the reads are separated by amplicon, and the unique barcodes in each amplicon are ordered according to the number of reads containing the barcode. The clustering procedure is based on our assumption that an error-free barcode is present in substantially more reads than any single erroneous version of the barcode. Given this assumption, barcodes that are within edit distance of 1 from each other are clustered as long as one of them has at least 6x as many reads as the other. Some exceptions are made for barcodes with a single reads and barcodes that are not of the expected length, allowing for more aggressive clustering of these barcodes with other barcodes. A detailed description of the clustering procedure is provided in Additional File 1.

### Building consensus reads

A consensus is generated for all the reads in each cluster based on the alignments of these reads to the reference genome. At each position in the reference genome, we use both the abundance and base quality scores to pick the consensus base and assign a base quality, using calculations very similar to those in [12].

### Variant calling from consensus reads

We analyzed the consensus reads with a standard pipeline that involves read alignment with BWA, post-processing of the alignments with GATK indel realigner, GATK base quality score recalibrator (BQSR), GATK base alignment quality (BAQ) computation [26], and trimming of the primer bases using custom scripts. We called variants using MuTect [27], with extended output enabled. We extracted the variants from the extended ouput by ignoring some or all of the following filters: dbSNP filter (most mutations in NA12878 are present in dbSNP), clustered position filter (because the reads are a product of amplicon sequencing), contamination, and fstar LOD.

Sensitivity is calculated as number of true positives (TP)/number of NA12878 unique SNVs (i.e. 134). False positive rate (FPR) is calculated as (number of false positives/29,343), where 29,343 is the NIST GIAB high-confidence target region.

### RNA amplicon analysis

To calculate the correlation of barcode counts to expected copies of ERCC RNA transcripts in the reaction, we followed the barcode extraction and clustering steps to derive the molecular barcode count for each observed amplicon in the sequencing reads. The barcode counts of 88 amplicons observed in all three sequencing runs were then normalized to a mean count of 10,000 and log2 transformed. The averages of three experiments were then plotted against the log2 transformed, expected copies of corresponding transcripts. The raw read count comparison was done similarly by first normalized to a mean read of 10,000, then log2 transformed and compared to the expected values. In order to calculate the CV of barcode assignment and PCR process, we first removed the variability in sequencer loading by slightly down sampling raw reads to the same levels (i.e. the minimum total reads of the three replicate runs). The barcode and read counts from down sampled data were used directly for CV calculation without any normalization or transformation steps. CV for the theoretical Poisson distribution is calculated as 1/sqrt(mean).

### Availability of supporting data

Primer sequences, additional figures, and tables are included in the additional files.

## Additional files

Additional file 1: **Supplementary materials.** Supplementary materials include Supplementary Tables and Figures. **Figure S1:** Two rounds of size selection purification efficiently removed unused BC primers and as a result eliminated any primer dimer problem. **Figure S2:** Molecular barcode efficiently removes PCR amplification noise. **Table S1:** Descriptions of FFPE samples used in the paper. (DOCX 200 kb)

Additional file 2: **List of all primers used in the paper.** (XLSX 57 kb)
