# Supplementary material for: Reducing amplification artifacts in high multiplex amplicon sequencing by using molecular barcodes
Source: BMC Genomics. 2015 Aug 7;16(1):589. doi: 10.1186/s12864-015-1806-8 (PMC4528782; doi:10.1186/s12864-015-1806-8)
Supplement: Supplementary file 1 — Supplementary materials. Supplementary materials include Supplementary Tables and Figures. Figure S1: Two rounds of size selection purification efficiently removed unused BC primers and as a result eliminated any primer dimer problem. Figure S2: Molecular barcode efficiently removes PCR amplification noise. Table S1: Descriptions of FFPE samples used in the paper. (DOCX 200 kb) [file 12864_2015_1806_MOESM1_ESM.docx]

**Supplementary Materials**

**Supplementary Figure S1.**

Two rounds of size selection purification efficiently removed unused BC primers and as a result eliminated any primer dimer problem. These electrophoresis results were generated using 20ng human gDNA input and DNA Amplicon Panel II containing more than 900 primer pairs.

1. Protocol with one round of size selection after the barcode assignment step and after the limited amplification step. A significant amount of primer dimers were seen.
2. Protocol with two rounds of size selection after the barcode assignment step and one round of size selection after the limited amplification step. Primer dimers were negligible.

**
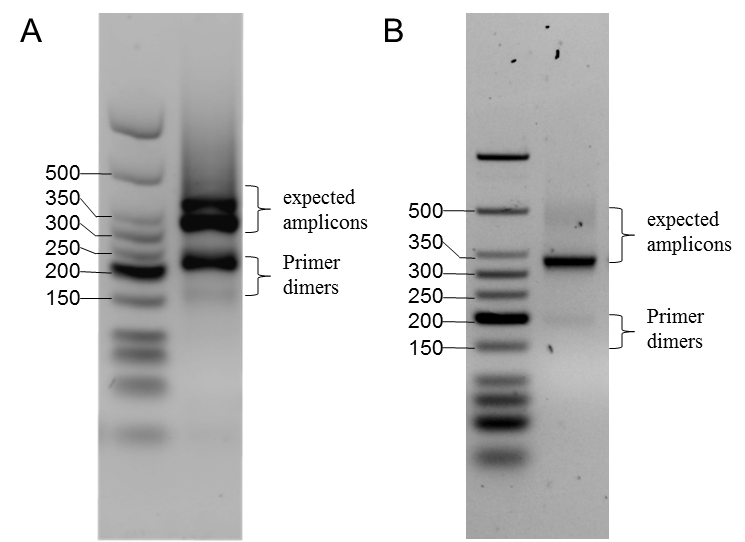
**

**Supplementary Figure S2. Molecular Barcode Efficiently Removes PCR Amplification Noise**

After step 4 of our protocol, the purified materials were split into three identical universal PCR amplifications. Since they were all coming off the same barcode assignment and enrichment step, these replicates can measure the technical variability of the universal PCR amplification. By comparing the magnitude of technical variance from replicate universal PCR amplifications with that from replicate barcode assignment reactions, we can estimate which step introduce the most variability in sequencing reads.

As in Fig 3b, most measurements using barcodes still have much smaller technical noises, than those using raw sequence reads. The technical noises were reduced by about 2.3-fold on average by using barcode counts. This magnitude is not much different from that in the replicate barcode assignment experiments. The major variability in read counts is likely introduced during universal PCR amplification.

**
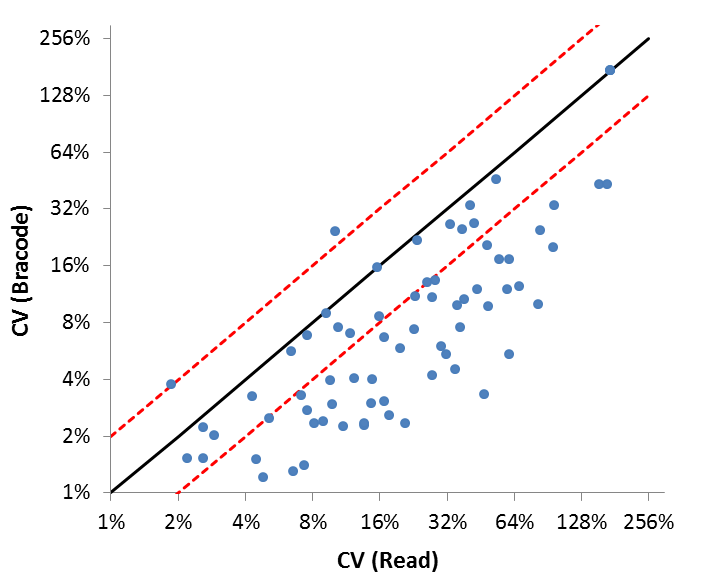
**

**Supplementary Table 1. Descriptions of FFPE samples used in the paper**

| **Sample ID** | T2 | T5 | LN2 | LT2 |
| --- | --- | --- | --- | --- |
| **Description** | Human gallbladder tumor, adenocarcinoma | Human breast tumor, invasive ductal carcinoma | Human match pair: lung, adjacent normal | Human match pair: lung, primary tumor |
| **Block Age** | 11yr | 10yr | 1yr | 1yr |
| **Cat. No.** | T2235118-2 | T2235086-2 | T823152-PN | T8235152-PT |
| **Lot No.** | A505211 | B506310 | B711351 | B711351 |
| **ng (by OD) / enrichment** | 105 | N/A | 40 | 51 |
| **ng (amplifiable) / enrichment** | 3.6 | 0.68 | 37.58 | 40.56 |
| **No. of universal PCR cycles used** | 25 | 28 | 22 | 22 |

The amount of amplifiable DNA is calculated based on the Ct value of a 200bp amplicon assay in the GeneRead DNA QuantiMIZE Kit. This assay is designed to target multiple copies of a 200bp region scattered across many chromosomes. The Ct values from FFPE DNA were compared to that from 5ng high quality human genomic DNA, in order to estimate PCR amplifiable DNA from the FFPE samples.

**Detailed description of the clustering procedure**

If the most frequent barcode has *n* reads, all barcodes that have more than 0.05x*n* reads are considered “real” barcodes (error-free copies of original molecular tags), as long as 0.05x*n* ≥ 3. All the remaining barcodes are considered “ambiguous”, in the sense that we are not yet sure whether these are initial molecular tags or erroneous copies of them. These “ambiguous” barcodes are processed in the order of decreasing read counts. Each “ambiguous” barcode is compared with each of the current “real” barcodes. If the “ambiguous” barcode has *q* reads, and a real barcode has *p* reads, and the edit distance between the two is 1, then the “ambiguous” barcode is merged with the “real” barcode if *p* ≥ 6*q*. If an “ambiguous” barcode has ≥2 reads and cannot be merged with any of the current “real” barcodes, then it is itself considered “real”, and we move on to the next “ambiguous” barcode. At the end of this procedure if we are left with any single-read ambiguous barcodes or irregular length ambiguous barcodes, they are merged with the most frequent “real” barcode that is within an edit distance of 2. Figure S3 shows an example of this clustering procedure in action.

TTTGTTAGTAAT 234

CGTGGGAGCGGA 201

TTTGTTAGTTAT 9

GGCTCGGTCCTG 5

CGTGGGAGTGGA 4

TGGGAGCGGA 3

TCAAAGTTCGGG 2

GCGCAGAAATCC 2

TTTATTAGTAAT 1

TTCGTTAGTAAT 1

TTTGTTAGTGAT 1

TTTTTAGTAAT 1

CGTGGGAGCGGG 1

CGTGGTAGCGGT 1

CGTGGAAGCGTA 1

GCTCGGTCCTG 1

GGCCCGGTCCTG 1

GCTCGGTCCTG 1

GGCCCGGTCCTG 1

**Step1: Count all unique barcodes**

**TTTGTTAGTAAT 234**

**CGTGGGAGCGGA 201**

TTTGTTAGTTAT 9

GGCTCGGTCCTG 5

CGTGGGAGTGGA 4

TGGGAGCGGA 3

TCAAAGTTCGGG 2

GCGCAGAAATCC 2

TTTATTAGTAAT 1

TTCGTTAGTAAT 1

TTTGTTAGTGAT 1

TTTTTAGTAAT 1

CGTGGGAGCGGG 1

CGTGGTAGCGGT 1

CGTGGAAGCGTA 1

GCTCGGTCCTG 1

GGCCCGGTCCTG 1

GCTCGGTCCTG 1

GGCCCGGTCCTG 1

**Step2: Barcodes with ≥0.05× [number of reads in the most frequent barcode] are real**

**TTTGTTAGTAAT 234**

TTTGTTAGTTAT 9

TTTATTAGTAAT 1

TTTGTTAGTGAT 1

TTCGTTAGTAAT 1

TTT-TTAGTAAT 1

**CGTGGGAGCGGA 201**

CGTGGGAGTGGA 4

CGTGGGAGCGGG 1

**GGCTCGGTCCTG 5**

-GCTCGGTCCTG 1

GGCCCGGTCCTG 1

**TCAAAGTTCGGG 2**

GCGCAGAAATCC 2

TGGGAGCGGA 3

CGTGGTAGCGGT 1

CGTGGAAGCGTA 1

**Step3: Cluster with edit distance of 1**

**TTTGTTAGTAAT 234**

TTTGTTAGTTAT 9

TTTATTAGTAAT 1

TTTGTTAGTGAT 1

TTCGTTAGTAAT 1

TTT-TTAGTAAT 1

**CGTGGGAGCGGA 201**

CGTGGGAGTGGA 4

CGTGGGAGCGGG 1

--TGGGAGCGGA 3

CGTGGTAGCGGT 1

CGTGGAAGCGTA 1

**GGCTCGGTCCTG 5**

-GCTCGGTCCTG 1

GGCCCGGTCCTG 1

**TCAAAGTTCGGG 2**

**GCGCAGAAATCC 2**

**Step4: Cluster single-read and irregular length barcodes with edit distance of 2**

**Figure S3:** Example of barcode clustering procedure in action. The numbers next to each barcode indicate the number of reads containing that barcode.

**Command line arguments for various steps in the analysis pipeline are listed below:**

Alignment: BWA-MEM 0.7.5a-r422

*bwa mem -M -L 1000,5 -t 8 ref.fasta cons_reads_R1.fq cons_reads_R2.fq | samtools view -Sb -F 256 -o reads01.bam –*

Indel Realignment: GATK Indel Realigner (GATKLite 2.3-9)

*java -XX:DefaultMaxRAMFraction=1 -XX:+UseParallelGC –jar GenomeAnalysisTKLite-2.3-9-gdcdccbb/GenomeAnalysisTKLite.jar -T RealignerTargetCreator -o realign.intervals -I reads01.bam --intervals ampliconInserts.bed -isr UNION --baq OFF -- --validation_strictness SILENT --interval_merging ALL -R ref.fasta -nt 8*

*java -XX:DefaultMaxRAMFraction=1 -XX:+UseParallelGC -jar GenomeAnalysisTKLite-2.3-9-gdcdccbb/GenomeAnalysisTKLite.jar -T IndelRealigner -I reads01.bam -o reads01.realigned.bam -LOD 5.0 --intervals ampliconInserts.bed -isr UNION --baq OFF --validation_strictness SILENT --interval_merging ALL –R ref.fasta -targetIntervals realign.intervals --disable_bam_indexing*

Base Quality Score Recalibration: GATK Recalibrator (GATKLite 2.3-9)

Note: In running the BQSR step on the simulated reads, it is necessary to provide the simulated variants as known variants, as all the variants in the simulated data appear near the ends of the reads.

*java -XX:DefaultMaxRAMFraction=1 -XX:+UseParallelGC -jar GenomeAnalysisTKLite-2.3-9-gdcdccbb/GenomeAnalysisTKLite.jar -T BaseRecalibrator -I reads01.realigned.bam -R ref.fasta --disable_indel_quals -knownSites simulated_variants.vcf -L primerPairs.bed -o reads01.recal.grp*

*java -XX:DefaultMaxRAMFraction=1 -XX:+UseParallelGC -jar GenomeAnalysisTKLite-2.3-9-gdcdccbb/GenomeAnalysisTKLite.jar -T PrintReads -I reads01.realigned.bam -R ref.fasta -BQSR reads01.recal.grp -o reads01.recal.bam -nct 8*

Base Alignment Quality Computation: GATK PrintReads (GATKLite 2.3-9)

*java -XX:DefaultMaxRAMFraction=1 -XX:+UseParallelGC -jar GenomeAnalysisTKLite-2.3-9-gdcdccbb/GenomeAnalysisTKLite.jar -T PrintReads -I reads01.recal.bam -R ref.fasta --baq RECALCULATE --baqGapOpenPenalty 30.0 -o reads01.baq.bam -nct 8*

Primer Trimming

*Custom python scripts using pysam library to trim away primer bases*

Variant Calling: MuTect (1.1.4)

*java -Xmx2g -jar /mnt/fdkbio04/home/rvijaya/software/muTect-1.1.4/muTect-1.1.4.jar --analysis_type MuTect --reference_sequence ref.fasta –intervals ampliconInserts.bed --input_file:tumor reads.trimmed.bam –out mutect_extended_stats.out --enable_extended_output --fraction_contamination 0.003 --minimum_mutation_cell_fraction 0.004 --min_qscore 17 --heavily_clipped_read_fraction 0.75 --downsample_to_coverage 1000000*
